# Forceful mastication activates osteocytes and builds a stout jawbone

**DOI:** 10.1038/s41598-019-40463-3

**Published:** 2019-03-20

**Authors:** Masamu Inoue, Takehito Ono, Yoshitaka Kameo, Fumiyuki Sasaki, Takashi Ono, Taiji Adachi, Tomoki Nakashima

**Affiliations:** 1 Department of Cell Signaling, Graduate School of Medical and Dental Sciences, Tokyo Medical and Dental University (TMDU), Tokyo, Japan; 2 Department of Orthodontic Science, Graduate School of Medical and Dental Sciences, Tokyo Medical and Dental University (TMDU), Tokyo, Japan; 3 Core Research for Evolutional Science and Technology (CREST), Japan Agency for Medical Research and Development (AMED), Tokyo, Japan; 4 Laboratory of Biomechanics, Department of Biosystems Science, Institute for Frontier Life and Medical Sciences, Kyoto University, Kyoto, Japan

## Abstract

Bone undergoes a constant reconstruction process of resorption and formation called bone remodeling, so that it can endure mechanical loading. During food ingestion, masticatory muscles generate the required masticatory force. The magnitude of applied masticatory force has long been believed to be closely correlated with the shape of the jawbone. However, both the mechanism underlying this correlation and evidence of causation remain largely to be determined. Here, we established a novel mouse model of increased mastication in which mice were fed with a hard diet (HD) to elicit greater masticatory force. A novel *in silico* computer simulation indicated that the masticatory load onto the jawbone leads to the typical bone profile seen in the individuals with strong masticatory force, which was confirmed by *in vivo* micro-computed tomography (micro-CT) analyses. Mechanistically, increased mastication induced Insulin–like growth factor (IGF)-1 and suppressed sclerostin in osteocytes. IGF-1 enhanced osteoblastogenesis of the cells derived from tendon. Together, these findings indicate that the osteocytes balance the cytokine expression upon the mechanical loading of increased mastication, in order to enhance bone formation. This bone formation leads to morphological change in the jawbone, so that the bone adapts to the mechanical environment to which it is exposed.

## Introduction

During biological activity, body tissues are subjected to various kinds of stimuli outside and inside the body. In order to adapt to such stimuli, body tissues remodel themselves. Bone tissue is remodeled *via* a repetitive cycle of osteoclastic bone resorption and osteoblastic bone formation on the bone surface. Osteoclasts are large, multinucleated cells that have capacity to degrade bone tissue^1,2^. Osteoblasts secrete bone matrix molecules, mostly collagen, and mineralize them to construct calcified tissue^3^. A portion of osteoblasts is embedded in the bone matrix and these cells differentiate into osteocytes. The balance between bone resorption and formation is strictly regulated by various biological systems, including the endocrine, nervous, immune and musculoskeletal system itself^1,2^.

During locomotion and exercise, bones are exposed to mechanical loading, one of the crucial triggering factors for bone remodeling that adapts the bones to the loading condition^3–5^. Exercise is known to increase bone mass and density; on the other hand, unloading causes a decrease in bone mass^6,7^. The loading onto the bone causes deformation of the bone matrix and fluid flow in the bone lacuna and canaliculi, in which osteocytes are embedded. Osteocytes are thought to sense such changes and respond to them by expressing cytokines^8,9^. We previously demonstrated that the osteocyte is an indispensable source of RANKL for osteoclast differentiation^10,11^. Osteocytes secrete Wnt1 and its inhibitor sclerostin. Sclerostin suppresses osteoblast differentiation and its expression is negatively correlated with the force loaded onto the bone^3^. IGF-1 is the most abundant growth factor in the bone matrix and promotes osteoblastogenesis^12^. It was shown that osteocytes highly express IGF-1 and its expression is upregulated by mechanical loading^13,14^. Thus, bone tissue is remodeled in response to mechanical loading, which is largely dependent on osteocytes and their expressed cytokines.

During the growth period, the jawbone is subjected to certain forces imposed by the surrounding tissues, including the teeth and oro-facial muscles (masticatory, lingual and mimetic muscles). Therefore, the resulting adult facial profile is believed to be closely associated with these forces, especially the ones generated by the masticatory muscles, which play a central role in food ingestion: a brachyofacial pattern (short face) in individuals with strong masticatory force and a dolicofacial pattern (long face) in individuals with weak^15^. Animal models of reduced mastication, induced by liquid or powder diets, recapitulate the phenotypes seen in humans^16,17^. However, it is unclear if masticatory force by itself is a sufficient cause in such skeletal phenotypes. The underlying cellular and molecular mechanisms remain elusive as well.

Here, we established a novel increased mastication mouse model by feeding mice with a HD, thus burdening maxillofacial tissues with a higher mechanical load. HD increased mastication frequency and enlarged the masseter muscles. Using a computer simulation, mechanical loading onto the lower jawbone (mandibular bone) by the masseter muscle, the masticatory muscle with the largest muscular force, was indicated to induce extrusion of the masseteric ridge and a shortening of the mandibular ramus. Micro-CT analysis showed that the extrusion and the shortening were more prominent in mice fed with the HD. In the extruded masseteric ridge of these mice, there was an increase of IGF-1 and a decrease of sclerostin in osteocytes, both of which are implicated in the promotion of bone formation. IGF-1 was shown to enhance osteoblastogenesis of the cells in the tendon.

These findings taken together indicate that the osteocytes in the mandibular bone increase IGF-1 and decrease sclerostin expression as the result of mechanical load. The alteration in the local cytokine milieu adjacent to the enthesis leads to bone formation, resulting in morphological change in the mandibular bone so the mechanical stress in the bone can be alleviated.

## Results

### Establishment of a novel increased mastication model

**Figure 1:**
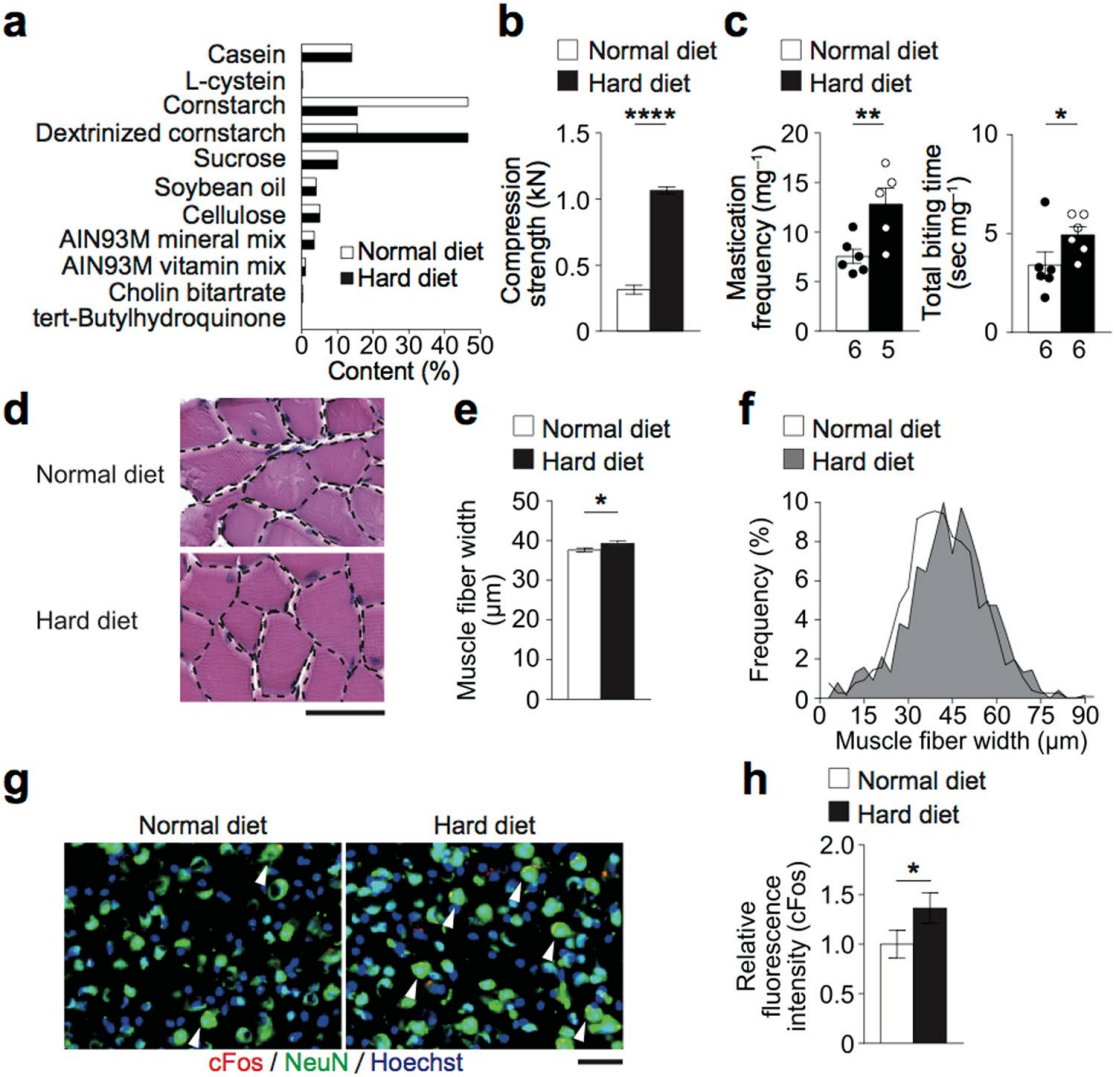
Establishment of a novel increased mastication model. Analyses of the hard diet and its effects on mice. (**a**) Nutrition components of the normal or hard diet (ND and HD, respectively). (**b)** Compression strength of the diets (n = 5 for each diet). (**c**) Mastication frequency and biting time. The number of masticatory cycles and total biting time required for 1 mg consumption of the diets. (**d**) Representative histological images of the masseter muscles of mice fed with the HD or ND stained with hematoxylin and eosin. Scale bar, 50 μm. (**e**) Minor axis width of the masseter muscle obtained from HE images using analysis software. In total, 750–800 muscle fibers per group were analyzed. (**f**) Histogram showing the distribution of the width of the masseter muscle fibers. (**g**) Representative Images of immunohistological staining of the primary motor cortex (M1) region of the cerebral cortex of HD– or ND–fed mice. The arrow heads indicate the NeuN^+^ neurons that expressed cFos. cFos (red); NeuN (green); and nucleus (blue). Scale bar, 100 μm. (**h**) Quantification of the neuronal activities as indicated by the fluorescence intensity of cFos. 2 sections per mouse and 4 mice each group were analyzed in the histological analyses. The number of biological replicates used in each animal experiment was shown under the corresponding bar. Student’s *t* test was conducted for statistical analysis. Error bars show the mean ± s.e.m. *p < 0.05; **p < 0.01; ****p < 0.0001.

In order to impose a higher mechanical load on the musculoskeletal tissue of the maxillofacial region, we developed a HD in which nutrition component is changed so that the hardness is greater than in a normal diet (ND) (Fig. 1a). The compression strength of the HD was approximately three times higher than that of a ND (Fig. 1b). The change did not result in any significant difference in food intake or body weight between the ND– and HD–fed mice (Supplementary Fig. 1a and b). Analyses on the bone and muscle of the limbs indicated that the difference between the diets did not cause either hypertrophy or atrophy in these tissues (Supplementary Fig. 2a–c**)**.

During the ingestion of the two diets, mastication frequency and time were significantly higher in the mice fed with the HD than the ND **(**Fig. 1c**)**. The fiber width of the masseter muscle, which is crucial for occlusion and mastication, was larger in the mice fed with the HD than the ND **(**Fig. 1d–f**)**. The neuronal activity of the primary motor cortex (M1) region of the cerebrum, which controls the masticatory muscles, was assessed according to the expression of cFos, as its expression is related to masticatory stimulation^17,18^. The M1 activity was higher in the HD–fed mice **(**Fig. 1g,h**)**. These findings indicate that the newly developed HD increased mastication and the mechanical load imposed on the maxillofacial region.

### Increased mastication results in bone formation at the enthesis of the masseter muscle

**Figure 2:**
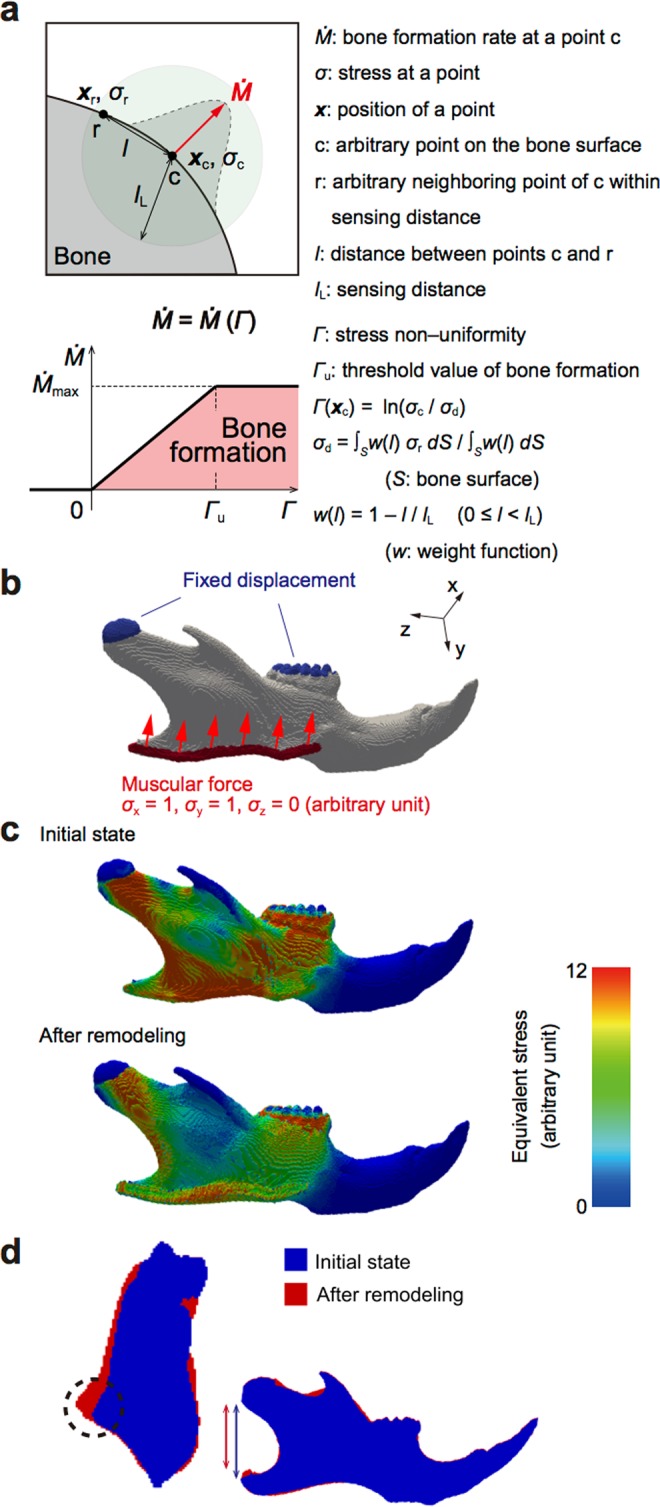
Computer simulation of the bone remodeling induced by mastication. A remodeling simulation of the voxel finite element (FE) model of the right mandibular bone (see Materials and Methods section for detail). (**a**) Schematics of the mathematical model, in which bone remodeling is driven by the stress non–uniformity, *Γ*. *Γ* at an arbitrary point c on the bone surface, *Γ*(***x***_c_), is expressed as the natural logarithm of the ratio of the stress at point c, *σ*_c_, to the weighted average surface stress within the sensing distance *l*_L_, *σ*_d_, *i.e*., $\Gamma ({{\boldsymbol{x}}}_{{\rm{c}}})=\,\mathrm{ln}({\sigma }_{{\rm{c}}}/{\sigma }_{{\rm{d}}})$. The bone formation rate $\dot{M}$ at the point c is described as a function of *Γ*: $\dot{M}=0$ when $\Gamma  < 0$; $\dot{M}=({\dot{M}}_{{\rm{\max }}}/{\Gamma }_{{\rm{u}}})\Gamma$ when $0\le \Gamma  < {\Gamma }_{{\rm{u}}}$; and $\dot{M}={\dot{M}}_{{\rm{\max }}}$ when $\Gamma \ge {\Gamma }_{{\rm{u}}}$, where *Γ*_u_ indicates the threshold value of bone formation. (**b**) Boundary conditions of the voxel FE model of the mandibular bone. Deformation of the condyle and molars was fixed (tinted blue). Muscular force was applied at the masseteric ridge (tinted red), antero–superio–laterally (red arrows). (**e**) Distribution of the mechanical stress in the FE model, before and after remodeling under the muscular force. (**d**) Superimposition of the images from FE models before and after remodeling. Left: coronal–section and right: lateral projection. The dotted circle indicates the extrusion of the masseteric ridge. The blue and the red lines denote the mandibular height.

Masticatory force is closely related to the shape of the bones in the maxillofacial region, especially the lower jawbone, *i.e*. the mandibular bone^15^. However, it is unclear if it is masticatory force itself that changes mandibular morphology. Thus, we predicted the morphological change of the mandibular bone under a mechanical loading condition, using a remodeling simulation. We adopted a computer model in which bone formation occurs at a point where the mechanical stress is higher than in the surrounding area^19,20^, so that the non-uniformity of the mechanical stress is reduced **(**Fig. 2a**)**. A voxel finite element (FE) model of the mandibular bone of a mouse was constructed and the muscular force of the masseter muscle was set to tract the masseteric ridge antero–superio–laterally **(**Fig. 2b**)**. At the initial state, stress distribution exhibited a non-uniformity, which was reduced after the bone remodeling **(**Fig. 2c**)**. In the mandibular bone after loading, there was a prominent extrusion of the masseteric ridge and a shortening of the mandibular ramus **(**Fig. 2d**)**.

**Figure 3:**
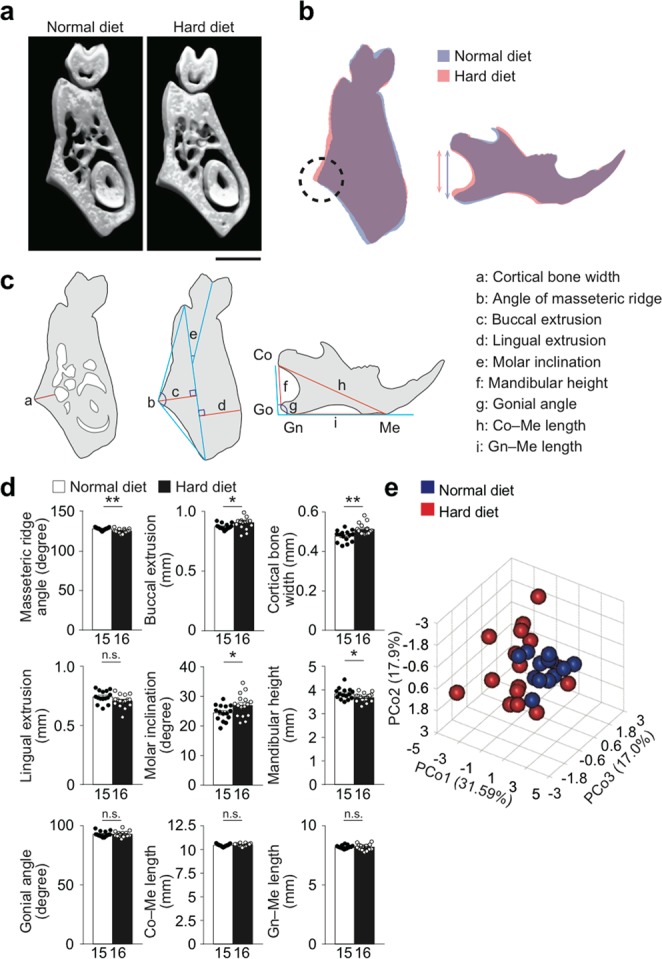
Increased mastication caused morphological change of the jawbone. Bone formation analyses in the increased mastication model. (**a**) Representative coronal micro-CT images of the mandibular bone of mice fed with the HD or ND. Scale bar, 1 mm. (**b**) Superimposition of the representative images of the mandibular bone of mice fed with the HD or ND. The dotted circle indicates the extrusion of the masseteric ridge. The blue and the red lines denote the mandibular height. (**c**) Schematics indicating the lengths and angles measured in the morphometric analysis. Condylion (Co), the most posterior point of the condylar head; Menton (Me), the most anterior and inferior point of the lower border of the mandibular bone; Gonion (Go), the most posterior point of the ramus; Gnathion (Gn), the most posterior and inferior point of the ramus; Gonial angle, angle between Co–Go and Gn–Me. (**d**) Angular and linear analyses of the mandibular bone (n = 15–16 per group). (**e**) Principal component analysis (PCA). 3 synthetic variables (PCo1–3) were generated from the 9 parameters obtained in (**d**) for dimension reduction and each mouse was plotted as a sphere (blue: ND–fed; red: HD–fed) in a three–dimensional space. The number of biological replicates used in each animal experiment was shown under the corresponding bar. Student’s *t* test was conducted for statistical analysis. Error bars show the mean ± s.e.m. *p < 0.05; **p < 0.01; n.s., not significant.

We then investigated if such morphological changes of the mandibular bone actually occur *in vivo* using micro-CT images of the mandibular bone. As predicted by the computer simulation **(**Fig. 2d), the masseteric ridge was more extruded and the mandibular ramus was shorter in the HD–fed than ND–fed mice (Fig. 3a,b). Angular and linear analyses further revealed the extrusion of the masseteric ridge to be related to the thickening of the cortical bone at the site (Fig. 3c,d). In addition, the lingual inclination of the molar was larger and the mandibular height was lower in the HD–fed mice than ND–fed mice (Fig. 3c,d). With the parameters obtained (Fig. 3d), principal component analysis (PCA) indicated that the increased mastication model mice exhibit a distinct mandibular morphology (Fig. 3e).

**Figure 4:**
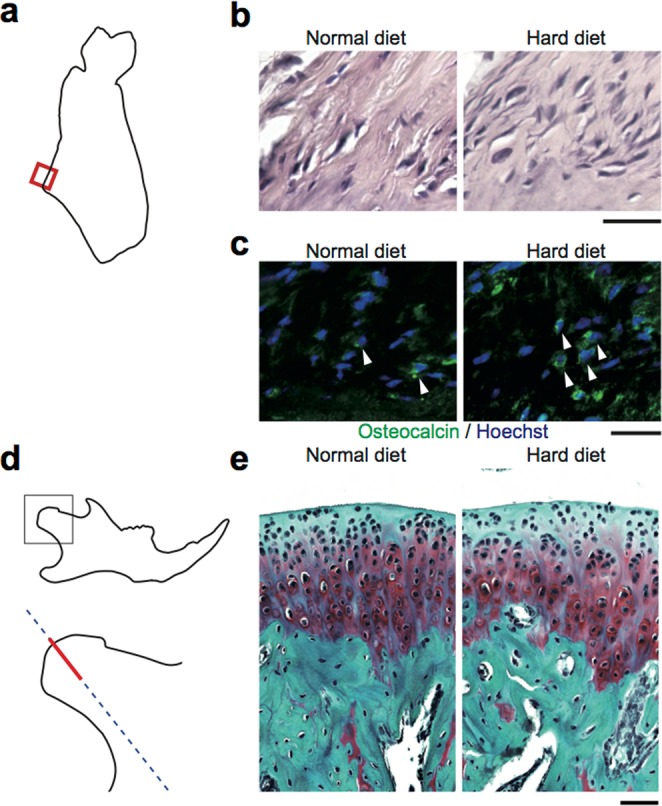
Increased mastication results in the bone formation at the enthesis of masseter muscle without affecting the condyle. (**a**) Schematic of the coronal section of the mandibular bone indicating the region of interest. Area specified by the red square was histologically analyzed. (**b**) Representative histological images of the coronal section of the mandibular bone at the enthesis stained with hematoxylin and eosin. Scale bar, 50 μm. (**c**) Representative immunohistological images of the coronal section of the mandibular bone at the enthesis. Scale bar, 25 μm. Osteocalcin (green); and nuclei (blue). (**d**) Schematic of the lateral image of the mandibular bone indicating the region of interest. The sections were sliced along the blue line and the area specified by the red bar was analyzed. (**e)** Representative histological images of the section of the condyle by Safranin O staining. Scale bar, 50 μm. 2 sections per mouse and 4 mice each group were analyzed in the histological analyses.

The extrusion of the masseteric ridge in the HD–fed mice prompted us to analyze the bone formation and resorption around the ridge, a site where the tendon of the masseter muscle inserts into the mandibular bone. Histological analyses showed that there was an increase in the number of osteocalcin–positive cells, osteoblasts, in the enthesis, while osteoclasts were undetectable (Fig. 4a–c). Although large masticatory force may be harmful on the condyle and result in the shortening of the ramus, there was no erosion on the surface of the condyle of mice fed with HD **(**Fig. 4d,e). No obvious differences in the cartilage and subchondral bone were observed **(**Fig. 4e), indicating that the increased mastication by the HD affects the mandibular bone morphology without damaging the cartilage in the condyle. Thus, increased mastication was revealed to remodel the mandibular bone by enhancing bone formation in the enthesis of the masseter muscle.

### Osteocytes produce IGF-1 in response to increased mastication, promoting osteoblastogenesis of tendon cells

**Figure 5:**
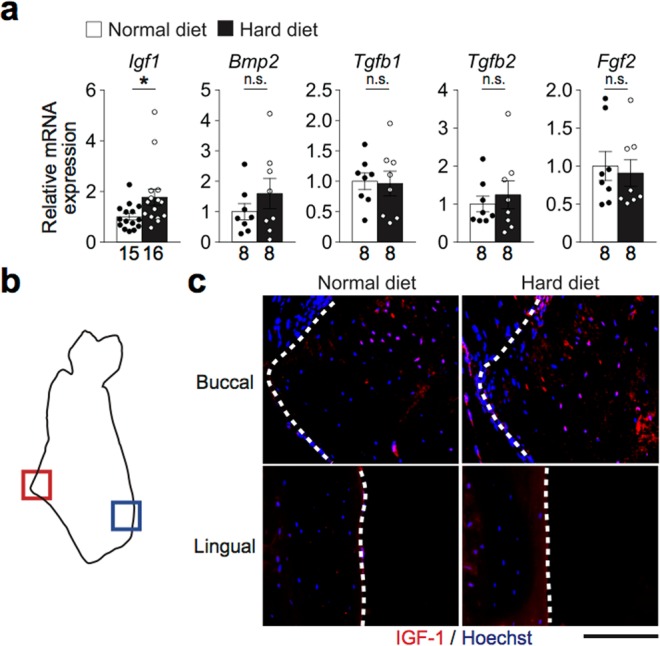
Osteocytes produce IGF-1 in response to increased mastication. Analyses of how increased mastication enhances bone formation. (**a**) mRNA expression of cytokines in the mandibular angle (n = 8–16 per group). (**b**) Schematic of the coronal section of the mandibular bone indicating the region of interest. The regions within the red and blue squares (the buccal and lingual cortical bones, respectively) were analyzed. (**c**) Representative immunohistological images of the masseteric ridge of mice fed with the HD or ND. IGF-1 (red); and nuclei (blue). The dotted lines indicate the bone surface. 2 sections per mouse and 4 mice each group were analyzed. Scale bar, 100 μm. The number of biological replicates used in each animal experiment was shown under the corresponding bar. Student’s *t* test was conducted for statistical analysis. Error bars show the mean ± s.e.m. *p < 0.05; n.s., not significant.

Next, we examined the mechanism by which increased mastication enhances bone formation. Because osteocytes are known to sense mechanical stress imposed on the bone and release cytokines^8,9^, we hypothesized that the osteocytes in the masseteric ridge would exhibit this activity. In search of such cytokines, we found a significantly higher expression of *Igf1* in the masseteric ridge of the HD–fed than ND–fed mice **(**Fig. 5a**)**. There was no significant difference in *Igf1* expression in the long bone **(**Supplementary Fig. 3**)**. Immunohistological analysis also showed that the protein level of IGF-1 was higher in the HD–fed mice, especially in the subsurface area of the masseteric ridge but not on the lingual side of the mandibular bone **(**Fig. 5b,c**)**. The expression of the IGF-1 receptor, *Igf1r*, in the masseteric ridge was comparable between the mice fed with the HD and ND **(**Supplementary Fig. 4**)**. Interestingly, in the masseter muscle, *Igf1r* expression was higher in the mice fed with the HD **(**Supplementary Fig. 4**)**, suggesting that osteocyte IGF-1 can induce muscle enlargement by increased mastication **(**Fig. 1d–f**)**.

**Figure 6:**
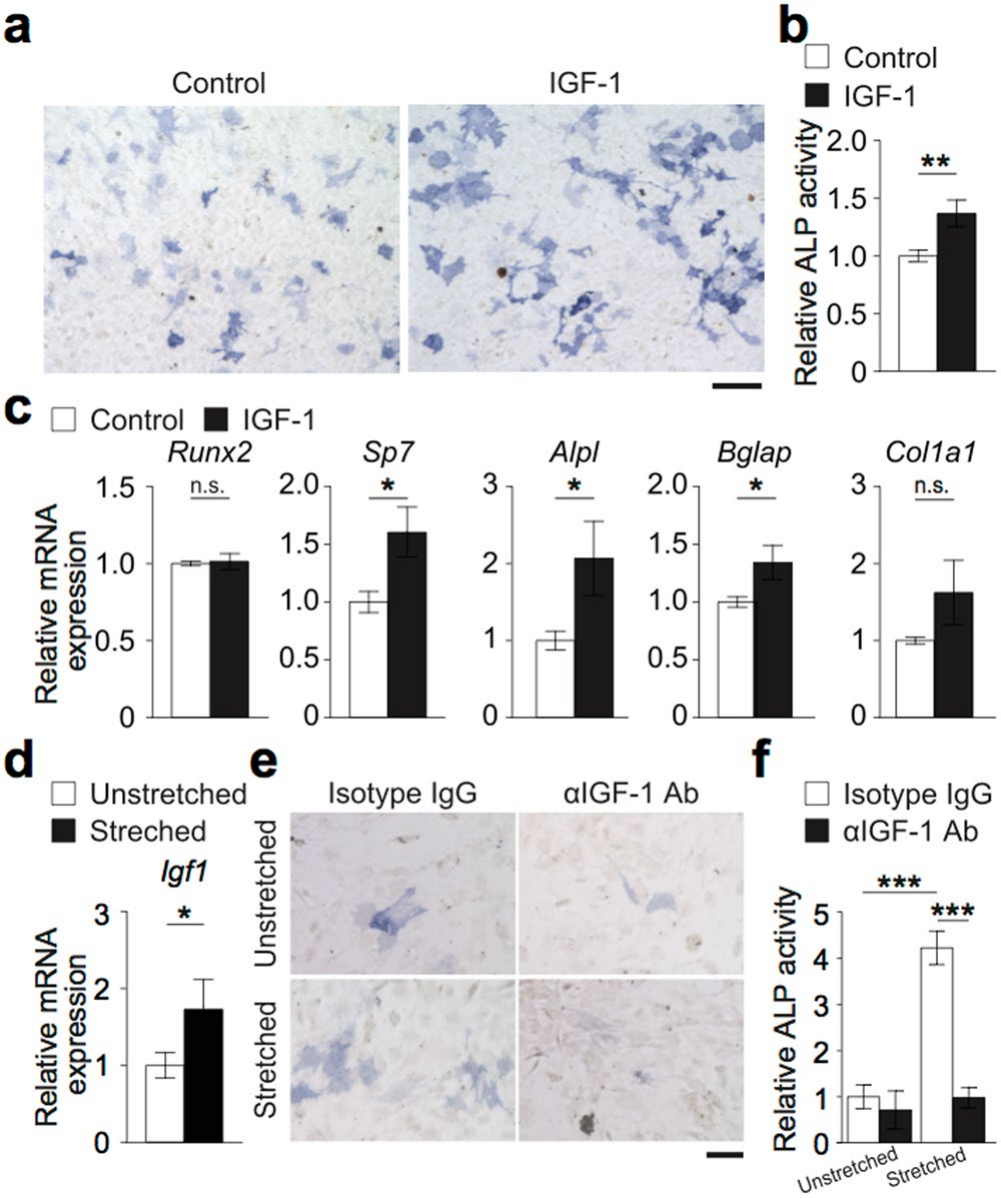
Osteocyte IGF-1 promotes osteogenesis of tendon cells. (**a**) *In vitro* osteoblast differentiation of tendon cells supplemented with IGF-1. Representative alkaline phosphatase staining images of the tendon cells on day 7 are shown. Scale bar, 100 μm. (**b**) Relative alkaline phosphatase activity calculated by the densitometry of the images. (**c**) mRNA expression of osteoblastic genes on day 7. (**d**) mRNA expression of IDG-SW3 cells with and without mechanical stimuli on day 21. (**e**) Representative alkaline phosphatase staining images of tendon cells on day 7. Scale bar, 50 μm. (**f**) Relative alkaline phosphatase activity. αIGF-1 Ab, anti–IGF-1 antibody. The data of the *in vitro* experiments were obtained from 3–4 independent experiments with replicates of 1–3 wells. Student’s *t* test and two–way analysis of variance (ANOVA) with Tukey’s multiple–comparison test were conducted for statistical analysis. Error bars show the mean ± s.e.m. *p < 0.05; **p < 0.01; ***p < 0.001; n.s., not significant.

Based on the findings described above, cells were collected from the tendon and cultured in an osteogenic medium in the presence of IGF-1. Osteoblastogenesis, as indicated by the activity of alkaline phosphatase, was enhanced by IGF-1 **(**Fig. 6a,b**)**. The expression of osteoblastic genes, including *Sp7*, *Alpl* and *Bglap1* (encoding Osterix, alkaline phosphatase and osteocalcin, respectively) was upregulated by IGF-1 **(**Fig. 6c**)**. In order to examine if mechanically–stimulated osteocytes function as a supplier of IGF-1 for tendon cells in the process of bone formation, another *in vitro* experimental system was established. An osteocyte cell line, IDG-SW3 cells were cultured in a stretch chamber and underwent cyclic stretching and compression stimulation (see Materials and Methods). This mechanical stimulation induced an upregulation of *Igf1* expression **(**Fig. 6d). Supernatant of these cells was collected and added to the tendon cell culture, either in the presence or absence of an anti–IGF-1 antibody. The addition of supernatant of mechanically–stimulated osteocytes enhanced the osteoblastogenesis of the tendon cells, which was cancelled by the anti–IGF-1 antibody (Fig. 6e,f). These results indicate that osteocytes produce IGF-1 in response to increased mastication to induce osteoblastogenesis of tendon cells.

### Increased mastication reduces sclerostin expression in osteocytes

**Figure 7:**
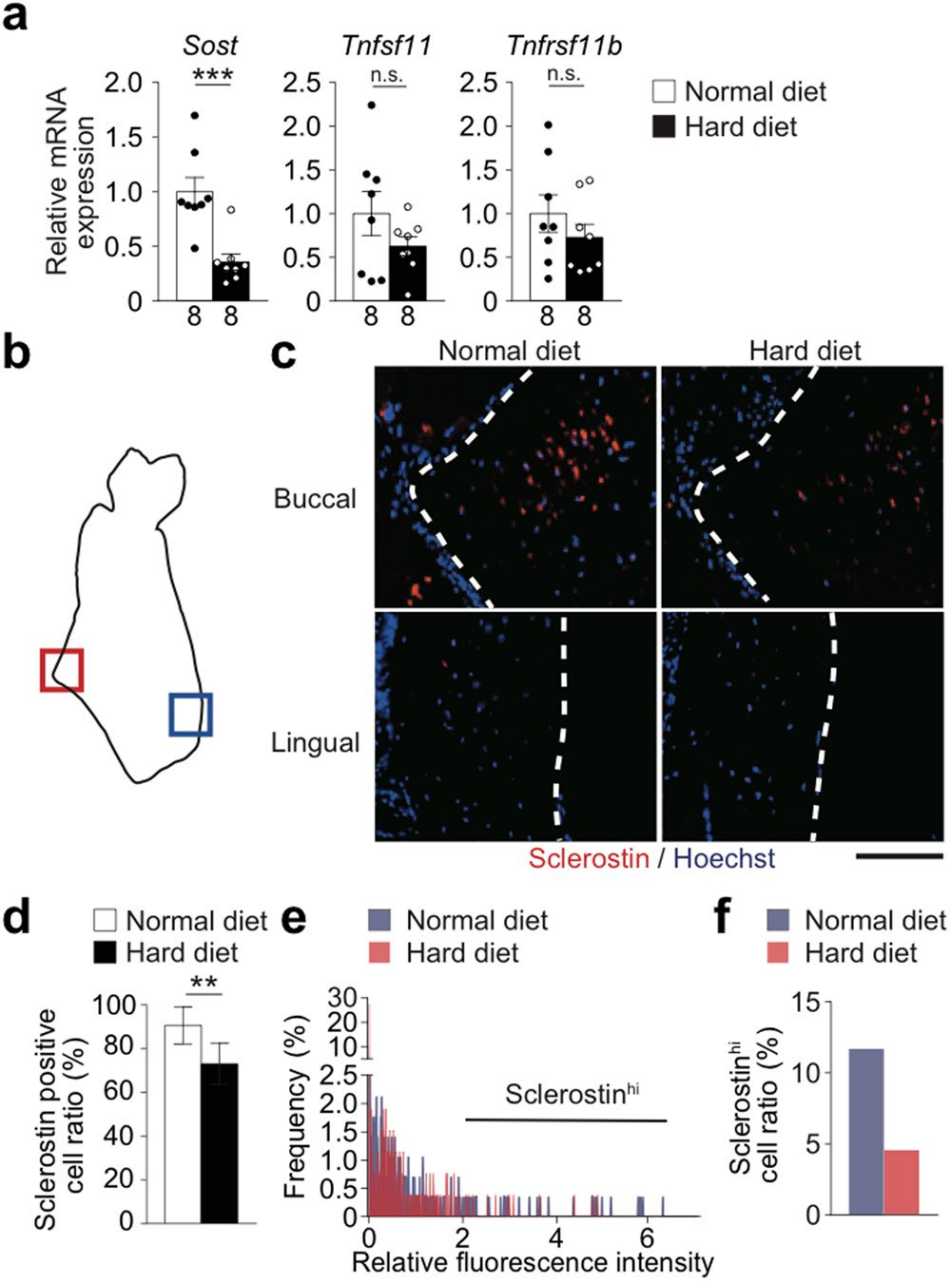
Increased mastication reduces sclerostin expression in osteocytes. Expression of the factors related to the suppression of bone formation and bone resorption. (**a**) mRNA expression of the cytokines in the mandibular angle (n = 8 per group). (**b**) Schematic of the coronal section of the mandibular bone indicating the region of interest. The regions within the red and blue squares (the buccal and lingual cortical bones, respectively) were analyzed. (**c**) Representative immunohistological images of the masseteric ridge of mice fed with the HD or ND. Sclerostin (red); and nuclei (blue). The dotted lines denote the bone surface. 2 sections per mouse and 4 mice each group were analyzed. Scale bar, 100 μm. (**d**) Sclerostin positive cell ratio calculated using the images obtained. (**e**) The distribution of the sclerostin expression intensity in individual cells, indicated by their fluorescence. In total, 200–250 cells per group were analyzed. (**f**) The proportion of osteocytes that highly express sclerostin. The number of biological replicates used in each animal experiment was shown under the corresponding bar. Student’s *t* test was conducted for statistical analysis. Error bars show the mean ± s.e.m. **p < 0.01; ***p < 0.001; n.s., not significant.

Osteocytes are known to produce the inhibitor of Wnt signaling sclerostin (encoded by the *Sost* gene), the expression of which is decreased by mechanical loading^21,22^. Thus, we analyzed its expression in the mandibular bone. The expression of *Sost* was significantly lower in HD–fed mice than in ND–fed mice **(**Fig. 7a**)**. There was no significant difference in the expression of *Tnfsf11* or *Tnfrsf11b* (encoding RANKL and OPG, respectively) **(**Fig. 7a**)**. There were no significant differences in the long bone **(**Supplementary Fig. 3**)**. In immunohistological analysis, sclerostin–positive osteocytes were detected in the inner compartment of the mandibular bone of both the HD–fed and ND–fed mice **(**Fig. 7b,c**)**. The ratio of sclerostin–expressing cells in the masseteric ridge was significantly lower in the HD–fed mice **(**Fig. 7d**)**. The frequency of osteocytes that highly expressed sclerostin (sclerostin^hi^ osteocytes), as indicated by high fluorescence intensity, was lower in the HD–fed mice **(**Fig. 7e,f**)**. In the lingual side, where morphological difference was not detected **(**Fig. 3d**)**, sclerostin expression was almost comparable between the mice fed with ND and HD **(**Fig. 7b,c). These findings indicate that increased mastication reduces the expression of sclerostin in the osteocytes, which can result in the promotion of bone formation.

## Discussion

“Adaptation to the environment” is a universal dogma in biological systems. In bone homeostasis, it is well known that bones under mechanical loading remodel themselves so that they become able to endure the loaded force. Variation in the facial profile is believed to be in part the result of differences in the force of mastication and occlusion^15^. However, the evidence for the causation was unclear, despite the apparent correlation. Although this correlation was well observed in experimental animal models^16,17,23–25^, the precise mechanism linking mechanical stimulation and mandibular morphology was unclear. The absence of a proper increased mastication animal model might have stagnated investigations on such mechanisms. In this study, we indicated that mechanical loading onto the jawbone itself can induce bone remodeling, using a novel hard diet. Furthermore, we found that the loading induces IGF-1 and suppresses sclerostin in osteocytes so as to enhance bone formation in the enthesis, leading to morphological change of the bone **(**Supplementary Fig. 5**)**.

Because bone remodeling is affected not only by mechanical loading but also many other stimuli inside and outside the body, it is extremely difficult to isolate the effect of mechanical load from those of other factors. Computer simulation is one of the means by which the effects of a single factor can be isolated: Our *in silico* remodeling simulation showed that a mechanical load imposed by the masseter muscle results in a bone phenotype similar to that of a human exhibiting strong occlusal force **(**Fig. 2c,d**)**, indicating masticatory force is a causative factor in different facial patterns. Subsequent *in vivo* micro-CT analysis revealed that increased mastication leads to a mandibular bone phenotype that highly resembles the one that was simulated **(**Fig. 3a–d**)**. With the theoretical rationale acquired by the simulation, our experimental results have become more reliable than results obtained by statistical analyses alone.

The jawbone is exposed to mechanical loading by mastication, occlusion and orthodontic forces. Because osteocytes are considered to act as a mechanosensor in bone, it has been speculated that these cells are active in jawbone remodeling. This study has underscored the significance of osteocytes by demonstrating that they upregulate the expression of IGF-1, which enhances bone formation in the enthesis so as to alleviate the disparity of the stress in the mandibular bone during mastication (Figs 2c, 5a–c).

Increased mastication suppressed sclerostin expression in the mandibular bone at the same time as an increase in IGF-1 expression **(**Figs 5–7**)**. It is reported in humans that the serum levels of these factors are negatively correlated^26^. It is also reported that osteocyte–specific *Igf1* deficiency relieves the suppression of sclerostin expression induced by mechanical loading, suggesting the regulation of sclerostin by IGF-1^27^, although the underlying mechanism has yet to be identified. The IGF-1 induced in osteocytes by increased mastication may regulate bone formation not only by enhancing osteoblastogenesis, but also by suppressing a bone formation inhibitor.

Clinicians have expected that masticatory force might serve a promising target in the treatment of skeletal anomalies such as skeletal open bite. However, there have been very few reports on clinical trials for such therapies^28,29^, possibly because of the lack of insight into their molecular basis. This study has indicated that masticatory force results in morphological change in the facial profile by modifying the function of osteocytes. Concerns about the adverse effects on the condyle might have distanced clinicians from such therapies. Our data suggests that increased mastication, within a certain extent, can alter the shape of the jawbone without damaging the condyle. Thus, there would be an opportunity for targeting masticatory force in the treatment and prevention of skeletal anomalies in the oro-maxillofacial region.

## Materials and Methods

### Experimental animals

C57BL/6 J mice 3 weeks of age were obtained from Clea Japan, Inc. These mice were maintained at Tokyo Medical and Dental University under specific pathogen–free conditions and fed with a normal diet (AIN93M: ND) or hard (AIN93M: HD) containing the same nutrition components except a modified cornstarch ratio. The body weight of the mice and their food intake were measured once a week. At 14 weeks of age, mice were sacrificed and the bone, muscle and brain tissues extracted were used for the subsequent analyses. The weight of the soleus muscle was measured after extraction. The number of the mice used in each experiment is described in the corresponding figure legend. All of the animal experiments were approved by the Institutional Animal Care and Use Committee and Genetically Modified Organisms Safety Committee of Tokyo Medical and Dental University (approval No. A2018-024A and 2015-007C, respectively) and conducted in accordance with the guidelines concerning the management and handling of experimental animals.

### Measurement of compression strength

The compression strength of the ND and HD was measured using a universal tester (Autograph AG-IS 5 kN, Shimazu). In the testing process, a pellet of ND or HD was set between 100 mm metal disks and underwent the compression process. The test was performed at the temperature of 23 ± 2 °C and humidity of 50 ± 5%. The maximum strength was measured during compression.

### Determination of mastication frequency

After fasting for 24 hours, a subject mouse was placed in a transparent box in which a ND or HD pellet was fixed on the wall. The mastication activity of the mouse was monitored and recorded for 5 minutes. The weight of the ingested pellet was expressed as the difference between the weights before and after the experiment. The number of mastication cycles and biting time were obtained from the recorded data, and the mastication frequency required for the ingestion of 1 mg pellet was calculated.

### Histological analyses of paraffin–embedded sections

Histological analyses of paraffin–embedded sections were performed as previously described^11,30^. Excised tissues were fixed by immersion in 4% paraformaldehyde (PFA) at 4 °C over night. Tissues containing bone further underwent decalcification in OSTEOSOFT (Merck Millipore) at 4 °C for 3 weeks. Fixed (and decalcified) tissues were dehydrated and embedded in paraffin. 6–μm–thick sections were cut.

Hematoxylin and eosin staining was carried out by staining deparaffinized sections with hematoxylin (Muto Pure Chemicals) for 3 minutes followed by 2 minutes of staining with eosin (Wako). The muscle fiber width (minor axis) was measured using measurement software (BZ–X analyzer, Keyence). The sections of the condyle were sequentially stained with Weigert’s iron hematoxylin solution (SIGMA) for 10 minutes, 0.05% Fast Green FCF solution (Wako) for 5 minutes and 0.1% Safranin O solution (Nacalai Tesque) for 5 minutes.

For immunohistological analyses of the expression of osteocalcin, IGF-1 and sclerostin in the mandibular bone and enthesis, deparaffinized sections were incubated with primary antibodies at 4 °C overnight: rabbit anti–osteocalcin antibody (1:100, TaKaRa); goat anti–IGF-1 antibody (1:40, R&D systems); and goat anti–sclerostin antibody (1:100, R&D systems). Sections were further incubated with secondary antibodies for 1 hour at room temperature: donkey anti-rabbit IgG Alexa Fluor (1:250, Invitrogen); chicken anti-goat IgG Alexa Fluor 594 (1:250, Thermo Fisher Scientific); and donkey anti-goat IgG Alexa Fluor 594 (1:250, Thermo Fisher Scientific). Nuclei were stained with Hoechst33342. The fluorescence intensity of sclerostin was analyzed using measurement software (BZ–X analyzer, Keyence).

### Histological analyses of frozen sections

Histological analyses of frozen sections were performed as previously described^17^. The excised brains underwent 4% PFA fixation overnight and followed by immersion in 30% sucrose for 3 days at 4 °C. Fixed tissues were embedded in optimal cutting temperature (O.C.T) compound (Sakura Finetek) and sliced into 20 μm–thick sections using a CM 3050S microtome (Leica Microsystems). Sections were incubated with primary antibodies at 4 °C overnight: mouse anti–NeuN antibody (1:100, Merck Millipore); and rabbit anti-cFos antibody (1:200, Merck millipore). They were then stained with secondary antibodies for 1 hour at room temperature: donkey anti–mouse IgG Alexa Fluor 488 (1:250, Thermo Fisher Scientific); and donkey anti-goat IgG Alexa Fluor 594 (1:250, Thermo Fisher Scientific). Nuclei were stained with Hoechst33342. The fluorescence intensity of cFos was analyzed using measurement software (BZ–X analyzer, Keyence).

### Micro-computed tomography (micro-CT) analyses of the bone

Micro-CT images were obtained with a micro-CT system (ScanXmate–D090S105) and Xsys software (Comscantecno)^17^. For the mandibular bone morphometric analysis, lateral images of the whole mandibular bone and coronal section images at the middle of the first molars were constructed and the parameters were calculated using ImageJ (National Institutes of Health). Principal component analysis was conducted using the web tool Easy PCA (http://hoxom-hist.appspot.com/pca.html). In the bone morphometric analysis of the femur, parameters were obtained using a 3–dimensional (3D) morphometry system (White Rabbit; Ratoc System Engineering).

### Mathematical model for bone remodeling

The mathematical model for bone remodeling simulation used in this study is based on the hypothesis that remodeling progresses so as to achieve a locally uniform mechanical state. This is a generic model to represent bone adaptation to mechanical loading, the validity of which has been shown previously^19,20^. In this model, bone remodeling was assumed to be driven by the local stress non-uniformity *Γ*, which is defined as the natural logarithm of the ratio of the stress at an arbitrary point on the bone surface, *σ*_c_, to the weighted average surface stress within the sensing distance *l*_L_, *σ*_d_.

### Incorporation into voxel based modeling

The mechanical stress (von Mises equivalent stress) in bone was numerically computed by means of a voxel finite element method (FEM). A voxel FE model of the mandibular bone was reconstructed from serial micro-CT images. The whole bone was discretized by using cubic voxel finite elements with an edge size of 37.2 μm. This voxel size is sufficiently small compared to the scale of mandibular bone that the dependency of simulated stress distribution on the voxel size is negligible. Because heterogeneous distribution of material properties does not drastically change the stress distribution, and therefore will not essentially influence bone morphological changes by remodeling, the bone was assumed to be a homogeneous and isotropic elastic material, with Young’s modulus *E* = 20 GPa and Poisson’s ratio *ν = *0.3.

In the framework of voxel modeling, the changes of bone morphology can be expressed by the removal/addition of voxel elements from/to the bone surface. Here, we considered only bone formation, *i.e*. the addition of voxel elements. Despite the fact that bone formation is discretely expressed, the rate of bone formation on the bone surface $\dot{M}$ can be described as the continuous function of *Γ* by taking $\dot{M}$ as the probability of bone formation. The following continuous function was introduced in order to describe the relationship between $\dot{M}$ and *Γ*: $\dot{M}=0$ when $\Gamma  < 0$; $\dot{M}=({\dot{M}}_{{\rm{\max }}}/{\Gamma }_{{\rm{u}}})\Gamma$ when $0\le \Gamma  < {\Gamma }_{{\rm{u}}}$; and $\dot{M}={\dot{M}}_{{\rm{\max }}}$ when $\Gamma \ge {\Gamma }_{{\rm{u}}}$, where *Γ*_u_ indicates the threshold value of bone formation. The parameters for remodeling simulation were set as follows: *l*_L_ = 1.12 mm, *Γ*_u_ = 0.6 and ${\dot{M}}_{{\rm{\max }}}=1$ (element/simulation step).

### *In vitro* osteoblastogenesis of tendon cells

The calcaneal, tail and masseteric tendons were dissected and cut into small pieces. The tissue fragments were digested with 0.1% collagenase (Wako) and 0.2% dispase II (GODO SHUSEI) at 37 °C with shaking at 150 rpm for 30 minutes. After filtration with a nylon filter, the cells were cultured and expanded for one to two days. The cells were plated on 48-well plate (3.0 × 10^4^ cells per well) and stimulated with an osteogenic medium (50 μg ml^−1^ ascorbic acid, 10 nM dexamethasone and 10 mM β-glycerophosphate in Dulbecco’s modified Eagle medium (DMEM)) supplemented with recombinant mouse IGF-1 (1 ng ml^−1^) 24 hours after the plating. Supernatant from mechanically–stimulated IDG-SW3 cells (see below) was added to the tendon cell culture at 25% of total volume, in which the cells were plated on 96-well plate (1.0 × 10^4^ cells in 100 μl per well). Either goat anti–IGF-1 antibody (1 μg ml^−1^, R&D systems) or control goat IgG (1 μg ml^−1^, R&D systems) was added to the culture.

Alkaline phosphatase staining was performed as previously described^30^. 7 days after the induction of osteoblastogenesis, cultured cells were fixed with 4% PFA for 15 minutes on ice. Alkaline phosphatase staining solution containing Napthol AS-MX phosphatase, 0.06 mg ml^−1^; N, N-dimethylformamide, 1%; and Fast blue BB salt, 1 mg ml^−1^ in 0.1 M Tris-HCl pH 8.0 was added after rinsing with PBS, and the cells were stained for 15 minutes at room temperature. The staining solution was washed away, and the stained cells were air dried. The color intensity was analyzed using measurement software (BZ–X analyzer, Keyence).

### Mechanical stimulation of IDG-SW3 cells

An osteocyte cell line, IDG-SW3 cells were maintained as previously described^31^. Cells were incubated in minimum essential medium α (MEM α) containing 10% FCS, 100 units ml^−1^ of penicillin, and 50 µg ml^−1^ of streptomycin on type I collagen (Corning) coated polydimethylsiloxane chambers (SC4Ha, Menicon Life Science) for 2 days at 33 °C. Osteogenic differentiation was induced in MEM α and 10% FCS supplemented with 50 μg ml^−1^ ascorbic acid and 4 mM β–glycerophosphate at 37 °C. On day 21 after differentiation, cells were subjected to mechanical loading using a cyclic unidirectional stretching device (ShellPa Pro, Menicon Life Science) as follows: stretch ratio, 5%; stretch frequency, 1 cycle per second; and stretch time, 4 hours. Total RNA and culture supernatant were collected after mechanical stimulation.

### Quantitative Reverse Transcriptase Polymerase Chain Reaction Analysis

The total RNA of the samples was extracted using a Maxwell RSC simply RNA Tissue Kit (Promega) according to the manufacturer’s instructions^17^. cDNA was synthesized with Superscript III reverse transcriptase (Thermo Fisher Scientific). Quantitative reverse transcriptase polymerase chain reaction analysis was performed with SYBR Green Realtime PCR Master Mix (TOYOBO) using a Light Cycler apparatus (Bio-Rad Laboratories). Gene expression was calculated with the ΔΔCt method and *Gapdh* expression was used for normalization. The primer sequences are listed below. *Gapdh*, 5′-ACCCAGAAGACTGTGGATGG-3′ and 5′-CACATTGGGGGTAGGAACAC-3′; *Igf1*, 5′-CTGGTGGATGCTCTTCAGTTCG-3′ and 5′-TGCTTTTGTAGGCTTCAGTGGG-3′; *Bmp2*, 5′-CAGGAAGCTTTGGGAAACAG-3′ and 5′-GTCGAAGCTCTCCCACTGAC-3′; *Tgfb1*, 5′-CCCTATATTTGGAGCCTGGA-3′ and 5′-CTTGCGACCCACGTAGTAGA-3′; *Tgfb2*, 5′-ATCGATGGCACCTCCACATATG-3′ and 5′-GCGAAGGCAGCAATTATGCTG-3′; *Fgf2*, 5′-CAACCGGTACCTTGCTATGA-3′ and 5′-TCCGTGACCGGTAAGTATTG-3′; *Runx2*, 5′-CCCAGCCACCTTTACCTACA-3′ and 5′-TATGGAGTGCTGCTGGTCTG-3′; *Sp7*, 5′-ATGGCGTCCTCTCTGCTTG-3′ and 5′-TGAAGGTCAGCGTATGGCTT-3′; *Alpl*, 5′-AACCCAGACACAAGCATTCC-3′ and 5′-GCCTTTGAGGTTTTTGGTCA-3′; *Bglap*, 5′-GCGCTCTGTCTCTGACCT-3′ and 5′-ACCTTATTGCCCTCCTGCTT-3′; *Col1a1*, 5′-GAGCGGAGAGTACTGGATCG-3′ and 5′-GTTAGGGCTGATGTACCAGT; *Sost*, 5′-TCCTGAGAACAACCAGACCA-3′ and 5′-CAGCTGTACTCGGACACAT-3′; *Tnfsf11*, 5′-AGCCATTTGCACACCTCAC-3′ and 5′-CGTGGTACCAAGAGGACAGAGT-3′; *Tnfrsf11b*, 5′-GTTTCCCGAGGACCACAAT-3′ and 5′-CCATTCAATGATGTCCAGGAG-3′; and *Igf1r*, 5′-GGAGAAGCCCATGTGTGAG-3′ and 5′-GTCGTGGATAACGAAGCCATC-3′.

### Statistical analysis

*P*-values were calculated using Student’s *t*-test or Tukey’s multiple–comparison test. Differences with a *p*-value of <0.05 were considered significant (*p < 0.05; **p < 0.01; ***p < 0.001; ****p < 0.0001; n.s., not significant, throughout the paper). Error bars show the mean ± standard error of the mean (s.e.m.). All statistical analyses were performed with GraphPad Prism 6.08 (GraphPad Software).

## Supplementary information

Supplementary figures

## Data Availability

No datasets were generated or analyzed during the current study.
