# Supplementary material for: Forceful mastication activates osteocytes and builds a stout jawbone
Source: Sci Rep. 2019 Mar 20;9:4404. doi: 10.1038/s41598-019-40463-3 (PMC6424982; doi:10.1038/s41598-019-40463-3)

SUPPLEMENTARY INFORMATION

Forceful mastication activates osteocytes and builds a stout jawbone

Masamu Inoue[1,2], Takehito Ono[1,3], Yoshitaka Kameo[4], Fumiyuki Sasaki[1,3], Takashi Ono[2], Taiji Adachi[3,4] and Tomoki Nakashima[1,3]

1. Department of Cell Signaling, Graduate School of Medical and Dental Sciences, Tokyo Medical and Dental University (TMDU)
2. Department of Orthodontic Science, Graduate School of Medical and Dental Sciences, Tokyo Medical and Dental University (TMDU)
3. Core Research for Evolutional Science and Technology (CREST), Japan Agency for Medical Research and Development (AMED)
4. Laboratory of Biomechanics, Department of Biosystems Science, Institute for Frontier Life and Medical Sciences, Kyoto University

Masamu Inoue and Takehito Ono contributed equally to this work

To whom correspondence may be addressed. E-mail: naka.csi@tmd.ac.jp.

Supplementary figure 1. Food intake and body weight were not affected by the hard diet

The effects of the novel hard diet on the mice throughout the study. **(a)** Food intake per week. **(b)** Body weight of mice fed with the HD or ND (n = 15–16 per group). Statistical analyses were conducted using Tukey's multiple-comparison tests. Error bars show the mean ± s.e.m. n.s., not significant.

Supplementary figure 2. The long bone and its related muscle were not affected by increased mastication

Analyses of the effects of the HD on the long bone and its related muscle. **(a)** Representative micro-CT images of the femur of mice fed with the HD or ND. Scale bar, 1 mm. **(b)** The bone volume and bone mineral density of the femur. **(c)** Soleus muscle weight per body weight (n = 15–16 per group). The number of biological replicates used in each animal experiment was shown under the corresponding bar. Statistical analyses were conducted using student's *t* test. Error bars show the mean ± s.e.m. n.s., not significant.

Supplementary figure 3. Gene expression in the long bone was not affected by increased mastication

Gene expression in the long bone of mice fed with the HD or ND (n = 4–8 per group). The number of biological replicates used in each animal experiment was shown under the corresponding bar. Student's *t* test was conducted for statistical analysis. Error bars show the mean ± s.e.m. n.s., not significant.

Supplementary figure 4. *Igf1r* expression is upregulated by increased mastication in the masseter muscle but not the masseteric ridge

Expression of *Igf1r* in the masseteric ridge and masseter muscle (n = 15–16 per group). The number of biological replicates used in each animal experiment was shown under the

corresponding bar. Statistical analyses were conducted using student's *t* test. Error bars show the mean ± s.e.m. *$p<0.05$. n.s., not significant.

Supplementary figure 5. Forceful mastication activates osteocytes and builds a stout jawbone

Graphical abstract. In order to consume the hard diet, the mastication force and frequency are increased by the enlargement of the masseter muscle, along with activation of the primary motor cortex (M1) region of the cerebral cortex. The masticatory force generated by the masseter muscle induces mechanical stress in the mandibular bone, leading to IGF-1 expression and sclerostin suppression in osteocytes. The upregulated IGF-1 may promote myogenesis in the masseter muscle *via* the IGF1R. IGF-1 induces osteoblastogenesis in the tendon cells to alter the mandibular bone morphology so that the disparity of the mechanical stress is alleviated.

Supplementary Figure. 1 Food intake and body weight were not affected by the hard diet

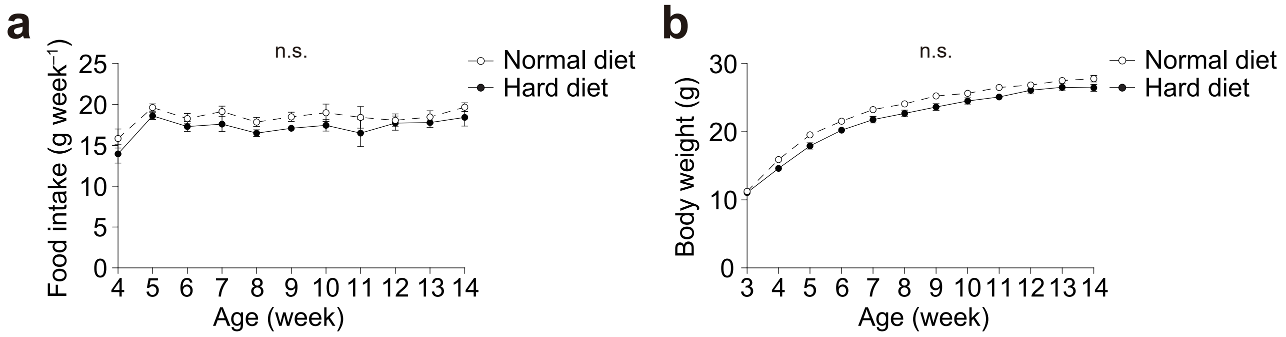

Supplementary Figure. 2 The bone structure of the femur was not affected by increased mastication

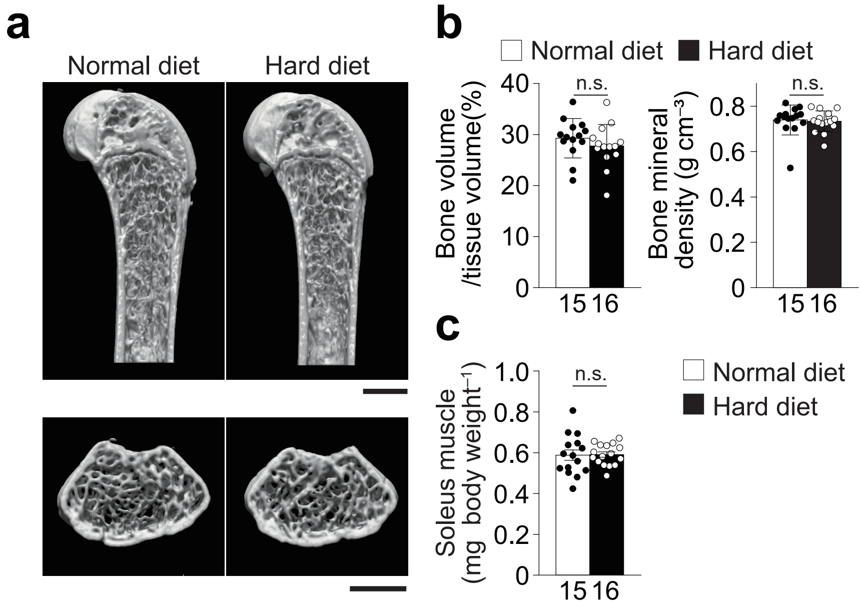

Supplementary Figure. 3 The gene expression of the femur was not affected by increased mastication

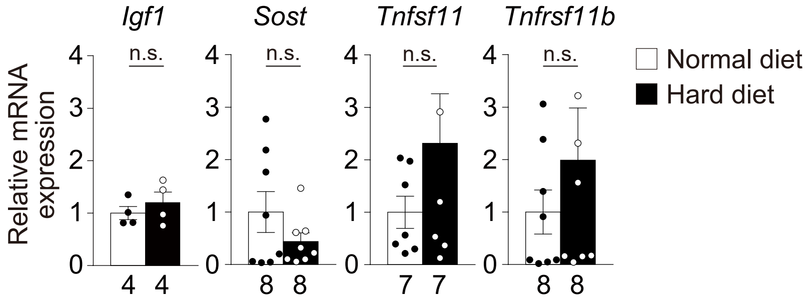

Supplementary Figure. 4 *Igf1r* expression is upregulated by the increased mastication in the masseter muscle but not in the masseteric ridge

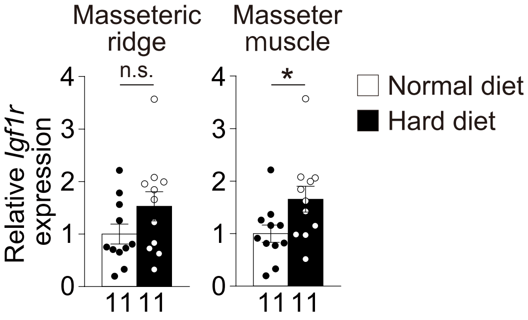

Supplementary Figure. 5 Forceful mastication activates osteocytes and builds a stout jaw bone

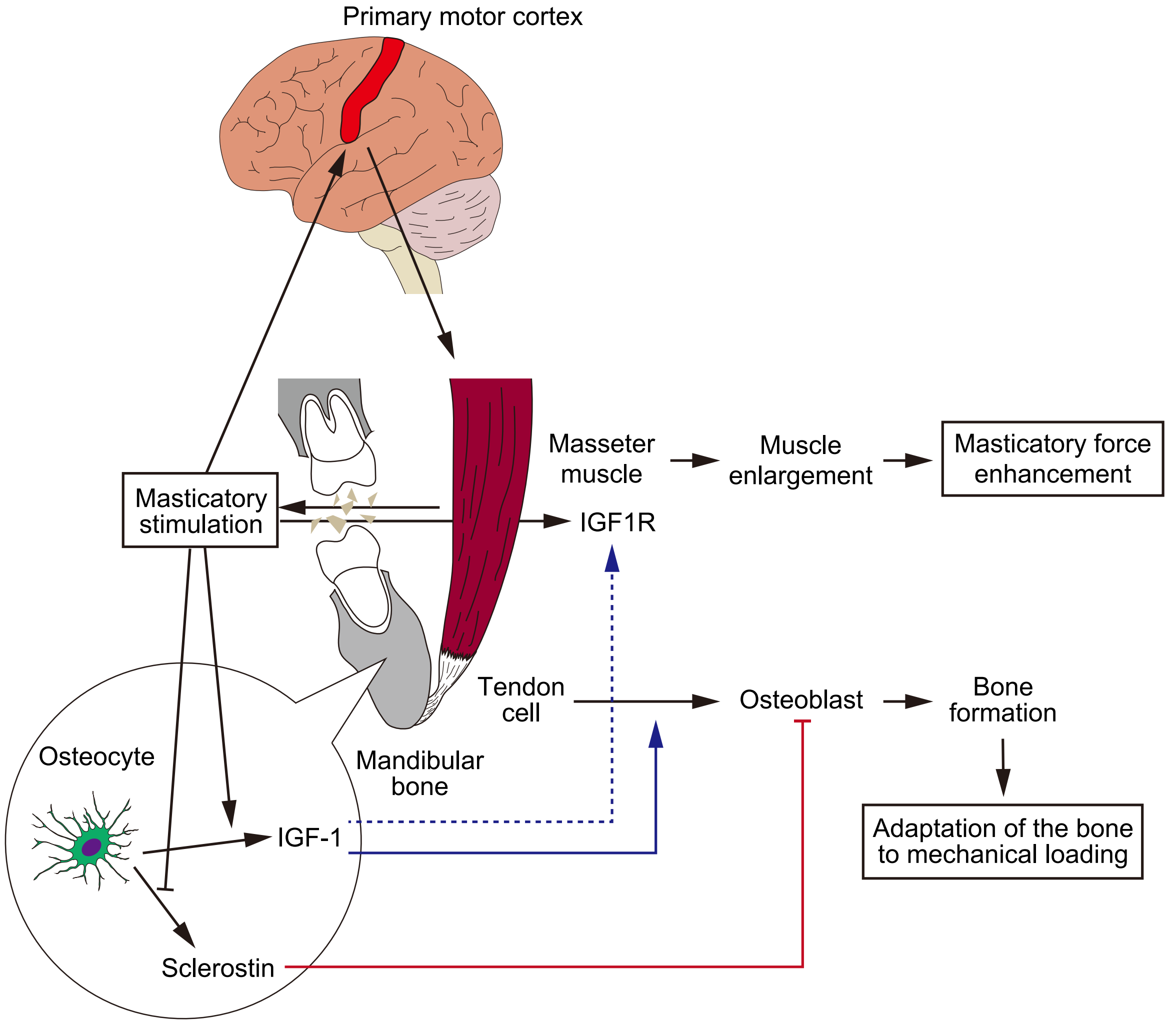

Supplement: Supplementary file 1 — Supplementary figures [file 41598_2019_40463_MOESM1_ESM.pdf]
